# Supplementary figures and images for: Antitumor Activities and Cellular Changes Induced by TrkB Inhibition in Medulloblastoma
Source: Front Pharmacol. 2019 Jun 26;10:698. doi: 10.3389/fphar.2019.00698 (PMC6606946; doi:10.3389/fphar.2019.00698)

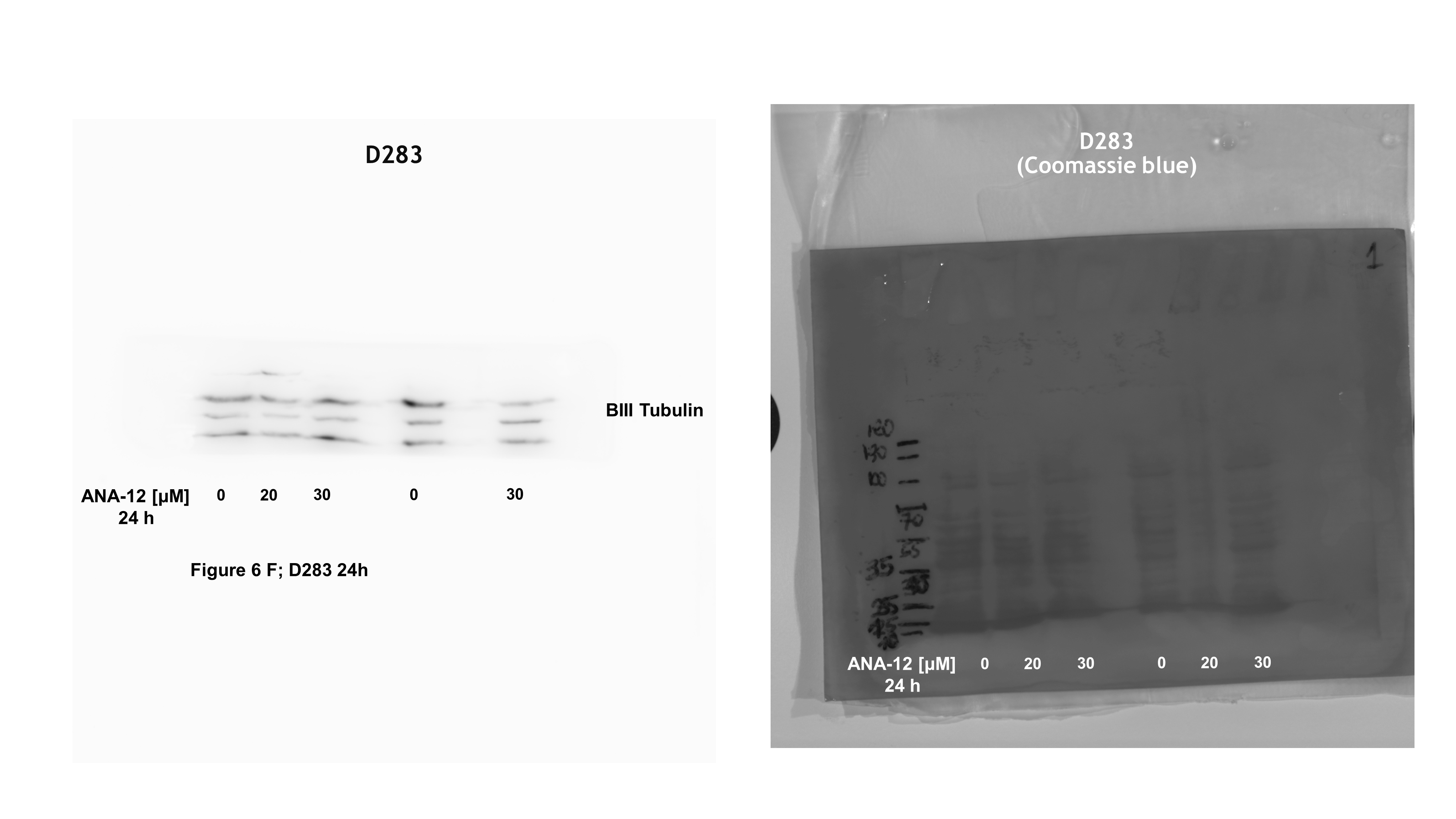

Supplement: Supplementary file 1 [file DataSheet_1.zip › Supplementary figures/WB-B-tubulin III D283 24h.tif]

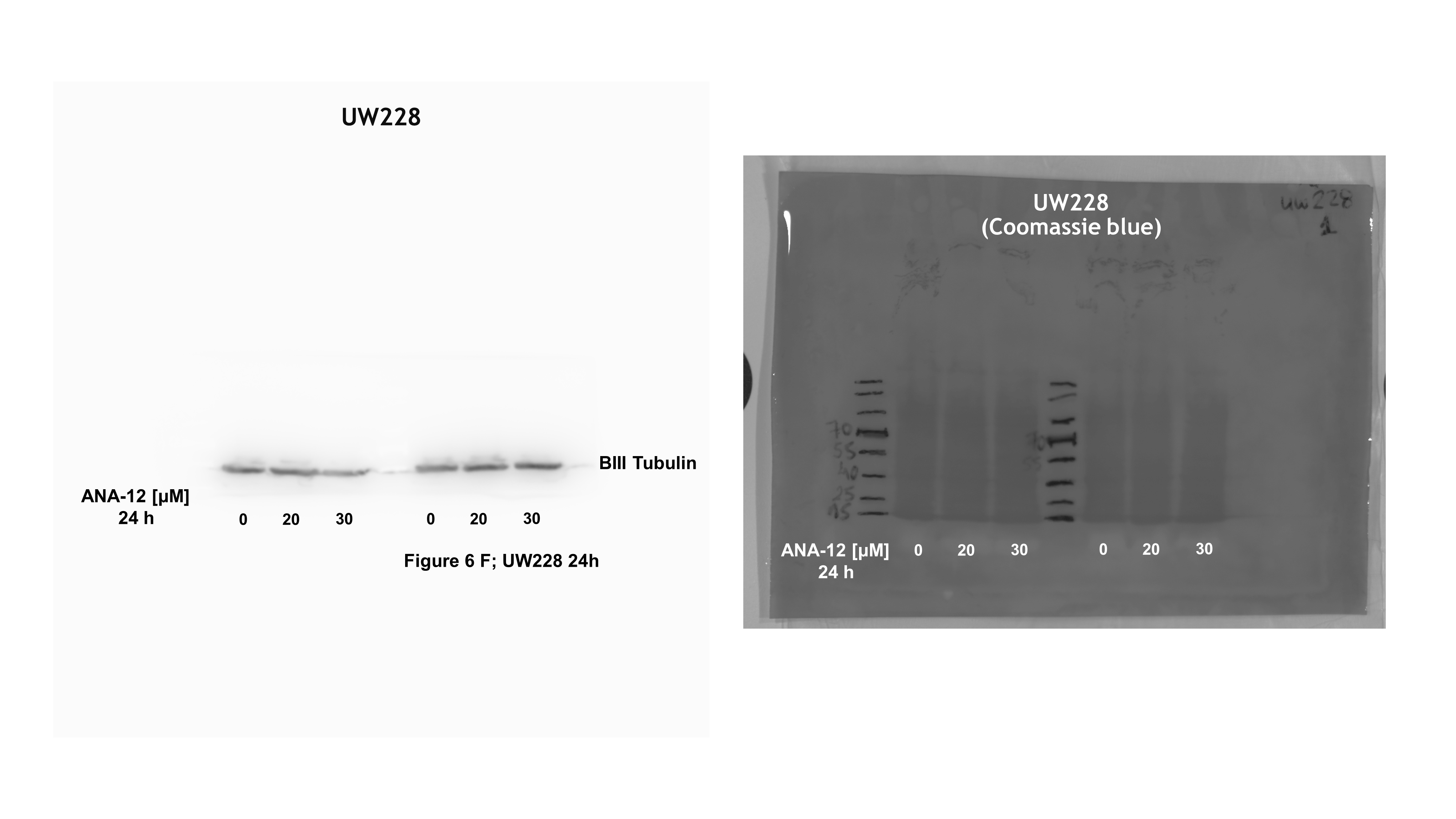

Supplement: Supplementary file 1 [file DataSheet_1.zip › Supplementary figures/WB-B-tubulin III UW228 24h.tif]

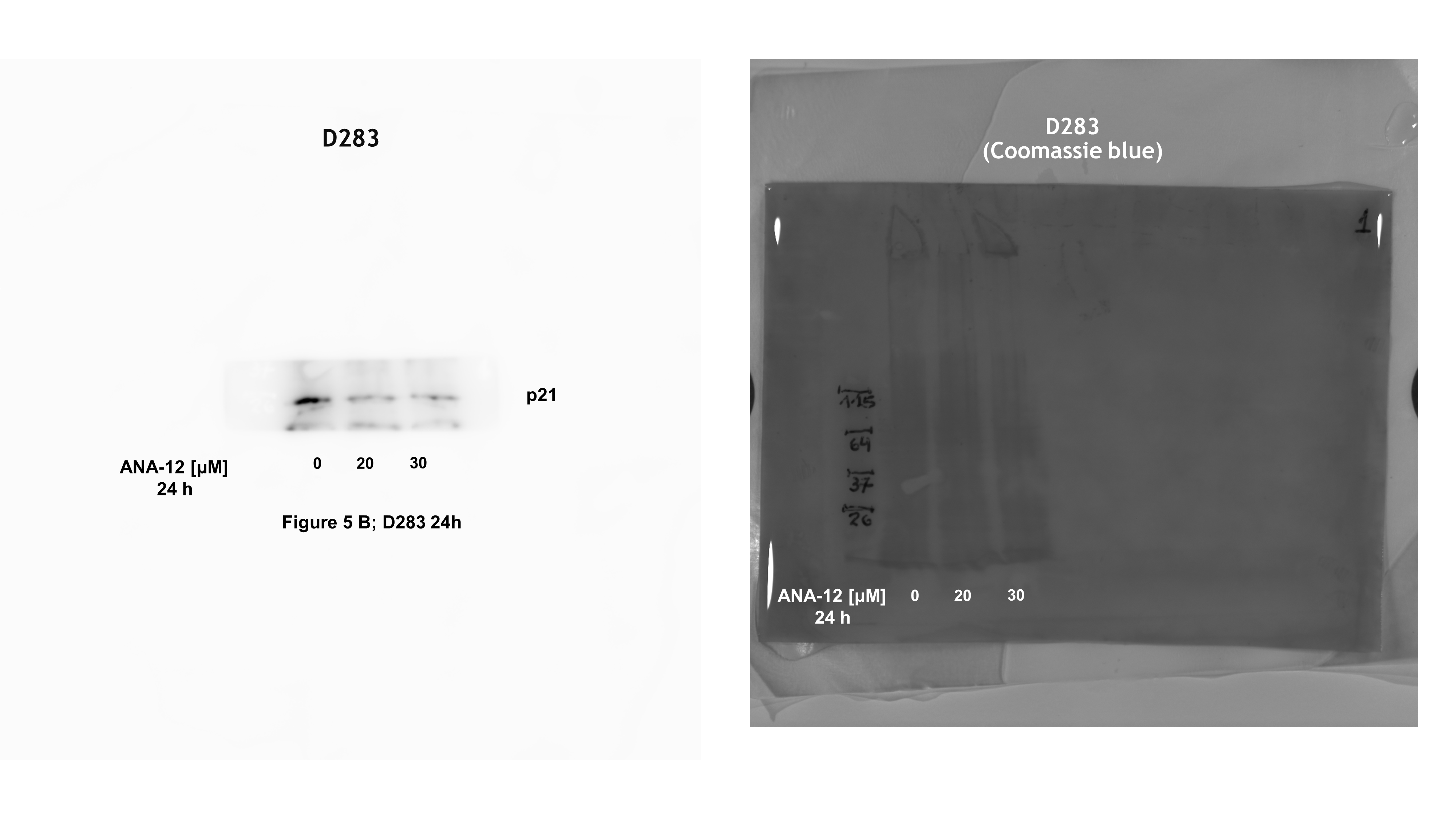

Supplement: Supplementary file 1 [file DataSheet_1.zip › Supplementary figures/WB-p21 D283 24h.tif]

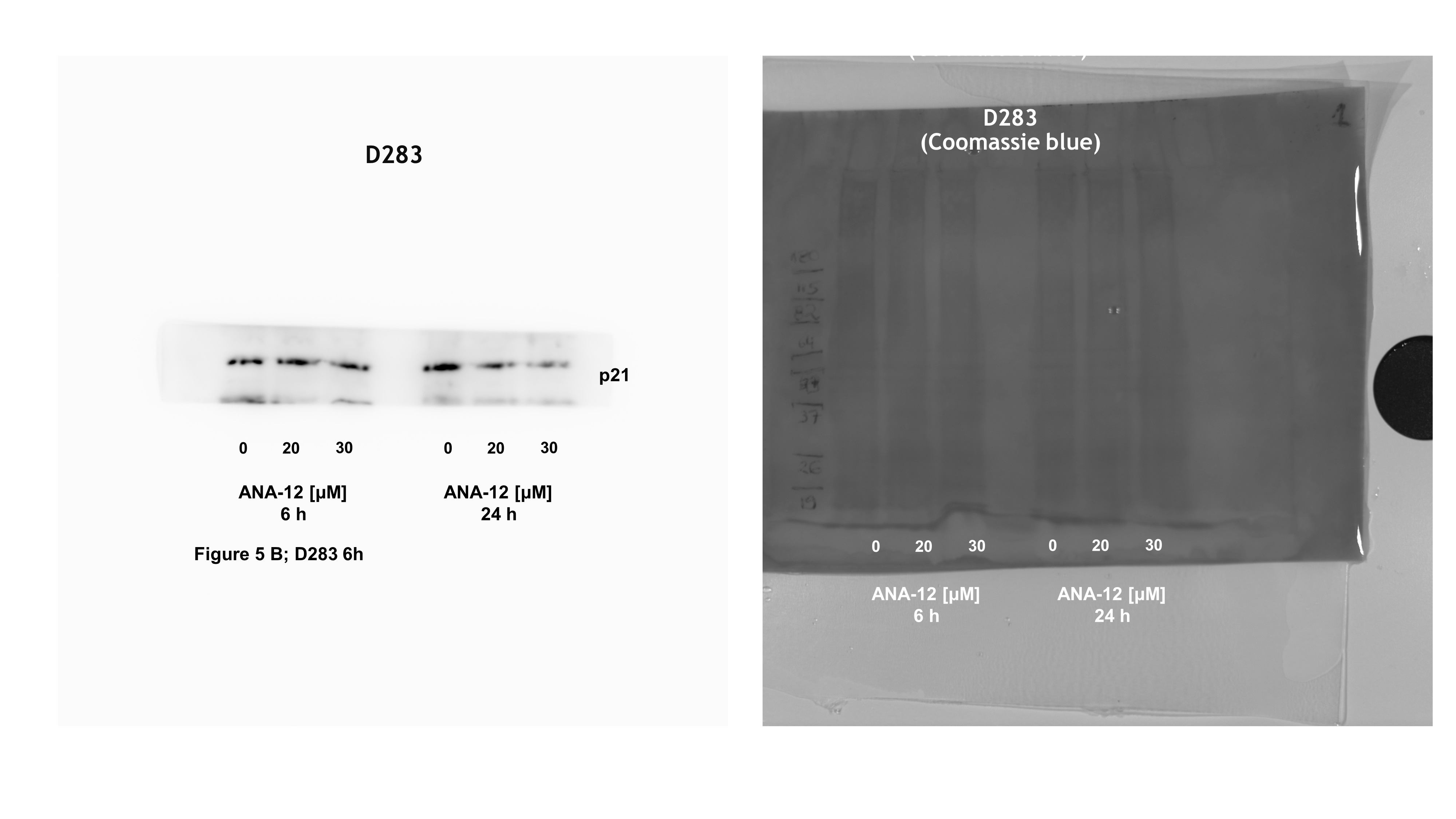

Supplement: Supplementary file 1 [file DataSheet_1.zip › Supplementary figures/WB-p21 D283 6h.tif]

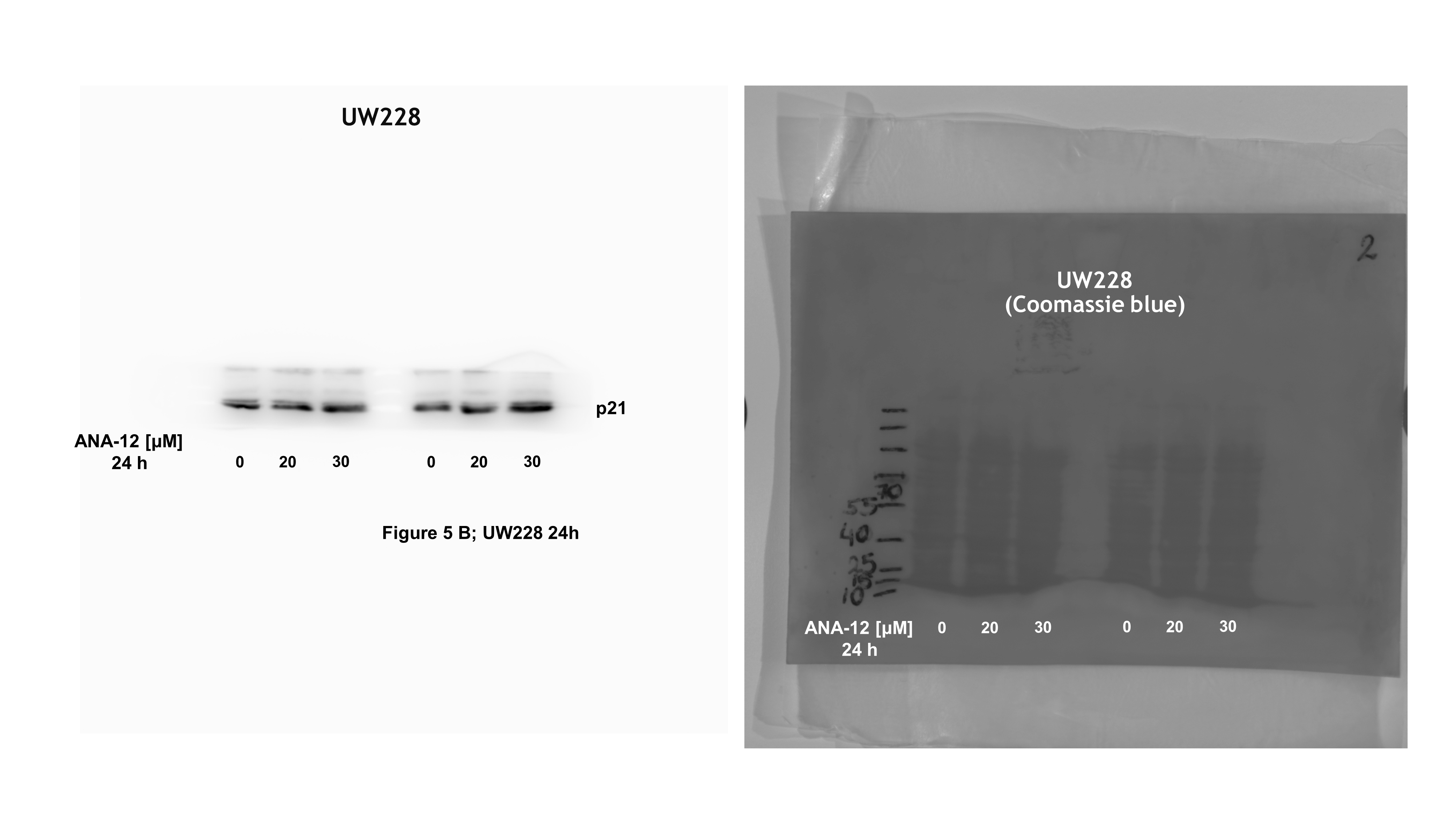

Supplement: Supplementary file 1 [file DataSheet_1.zip › Supplementary figures/WB-p21 UW228 24h.tif]

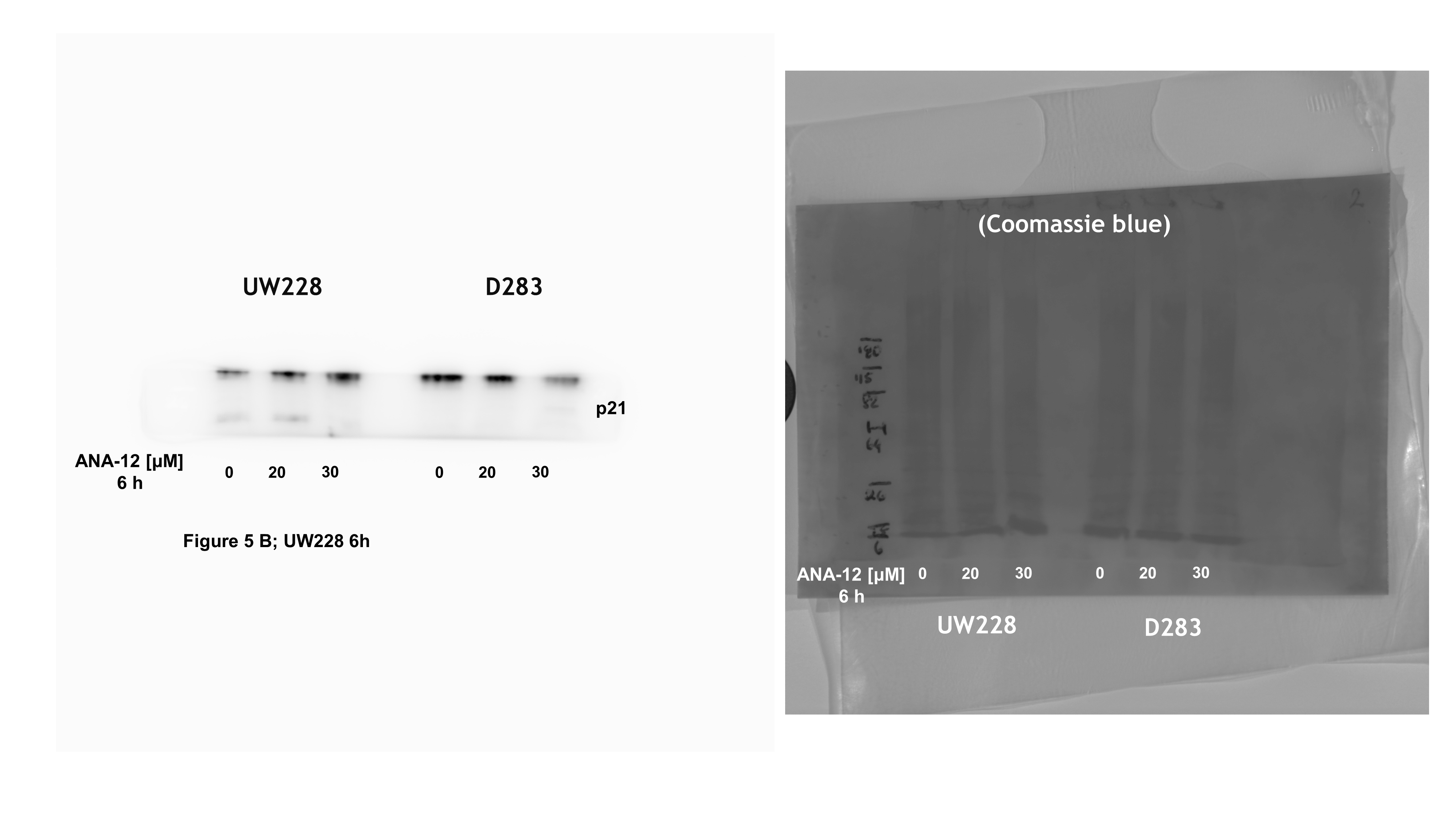

Supplement: Supplementary file 1 [file DataSheet_1.zip › Supplementary figures/WB-p21 UW228 6h.tif]
