# Supplementary material for: Antitumor Activities and Cellular Changes Induced by TrkB Inhibition in Medulloblastoma
Source: Front Pharmacol. 2019 Jun 26;10:698. doi: 10.3389/fphar.2019.00698 (PMC6606946; doi:10.3389/fphar.2019.00698)

Supplementary Material for:

Antitumor Activities and Cellular Changes Induced by TrkB Inhibition in Medulloblastoma

Amanda Thomaz* [1,2] • *Kelly de Vargas Pinheiro* [1,2] • *Bárbara Kunzler Souza* [1,2] • *Lauro Gregianin* [1,4,5] • *Algemir L. Brunetto* [1,3] • *André T. Brunetto* [1,3] • *Caroline Brunetto de Farias* [1,3] • *Mariane da Cunha Jaeger* [1,3] • *Vijay Ramaswamy* [6,7] • *Carolina Nör* [6,8] • *Michael D. Taylor* [6,7,8,9,10] • *Rafael Roesler* [1,2]

[1]Cancer and Neurobiology Laboratory, Experimental Research Center, Clinical Hospital (CPE-HCPA), Federal University of Rio Grande do Sul, Porto Alegre, RS, Brazil, [2] Department of Pharmacology, Institute for Basic Health Sciences, Federal University of Rio Grande do Sul, Porto Alegre, RS, Brazil, [3] Children's Cancer Institute, Porto Alegre, RS, Brazil, [4] Department of Pediatrics, School of Medicine, Federal University of Rio Grande do Sul, Porto Alegre, RS, Brazil, [5] Pediatric Oncology Service, Clinical Hospital, Federal University of Rio Grande do Sul, Porto Alegre, RS, Brazil, [6] The Arthur and Sonia Labatt Brain Tumour Research Centre, The Hospital for Sick Children, Toronto, ON, Canada, [7] Division of Haematology/Oncology, The Hospital for Sick Children, Toronto, ON, Canada,[8] Developmental and Stem Cell Biology Program, The Hospital for Sick Children, Toronto, ON, Canada, [9] Department of Laboratory Medicine and Pathobiology, University of Toronto, Toronto, ON, Canada, [10] Division of Neurosurgery, The Hospital for Sick Children, Toronto, ON, Canada.

**** Correspondence:***

Rafael Roesler, Department of Pharmacology, Institute for Basic Health Sciences, Federal

University of Rio Grande do Sul, Rua Sarmento Leite, 500 (ICBS, Campus Centro/UFRGS), 90050-170 Porto Alegre,RS, Brazil.

Telephone: +5551 33083183; fax: +5551 33083121.

E-mail: rafaelroesler@hcpa.edu.br

SUPPLEMENTARY FIGURE S1 Original Western blots membranes analysed by ImageQuant LAS500 (GE Healthcare Life Sciences, Little Chalfont, UK) and original membranes stained with Coomassie blue (0.025%) for western blots in Figures 5 and 6. Membranes were cut prior to antibody stainings to allow for detection of proteins running at different sizes on the same membrane.

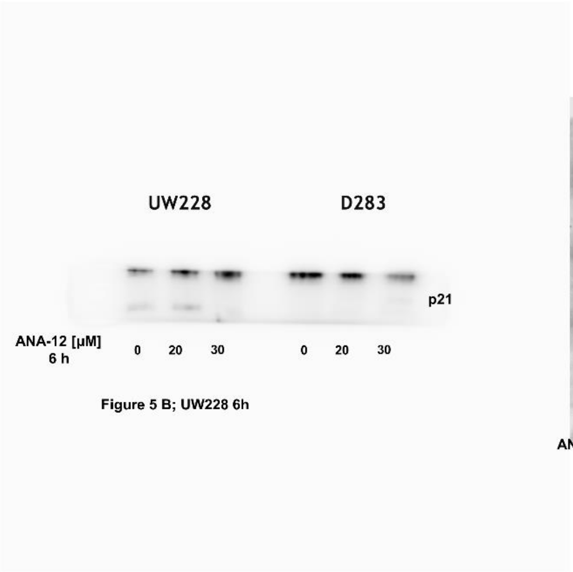

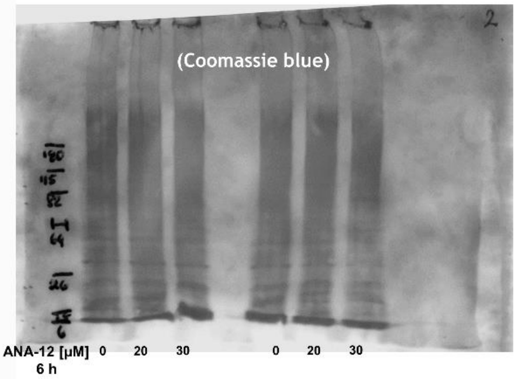

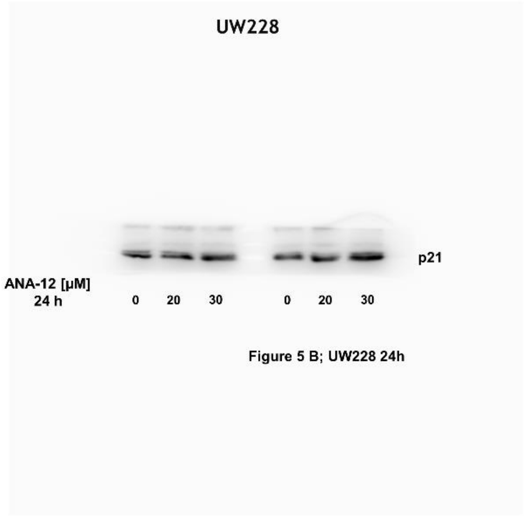

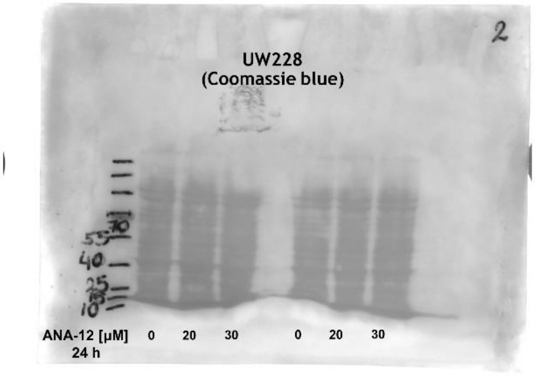

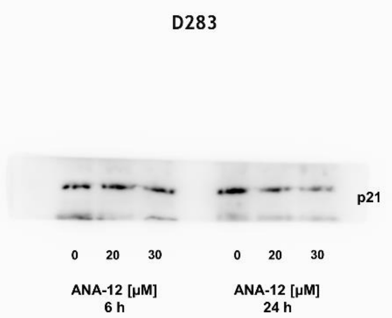

Figure 5 B; D283 6h

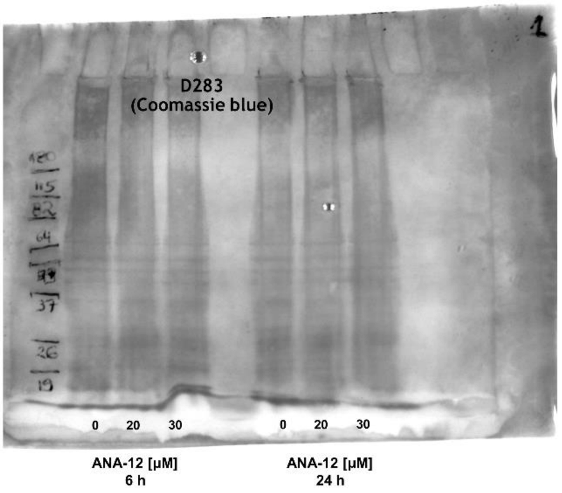

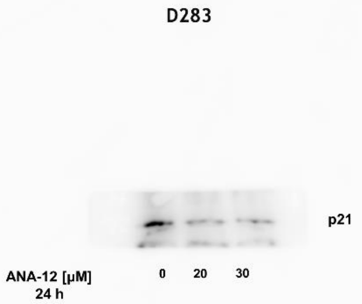

Figure 5 B; D283 24h

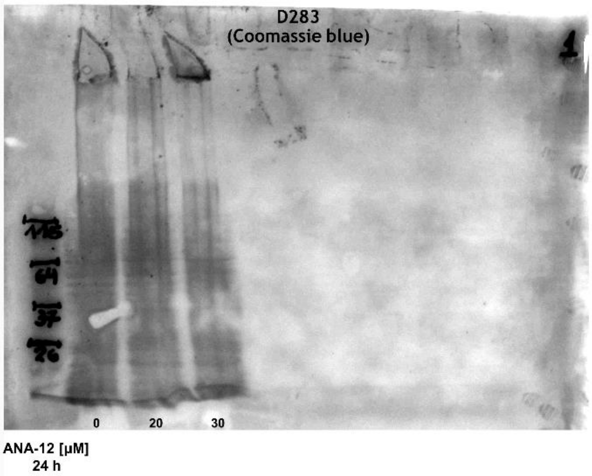

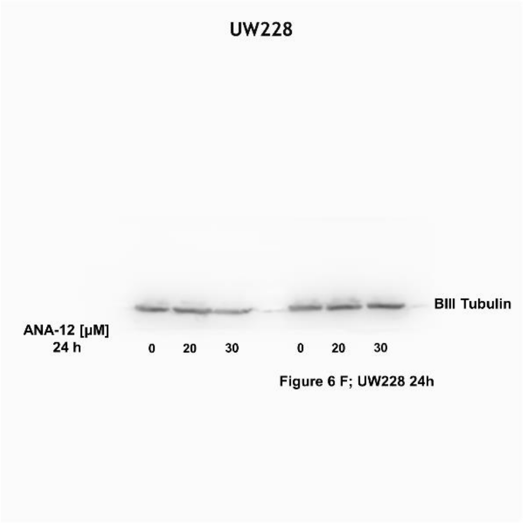

Figure 6 F; UW228 24h

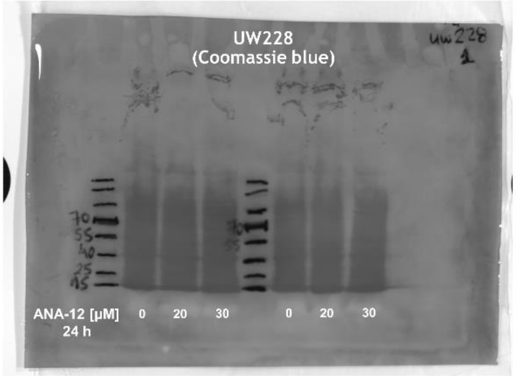

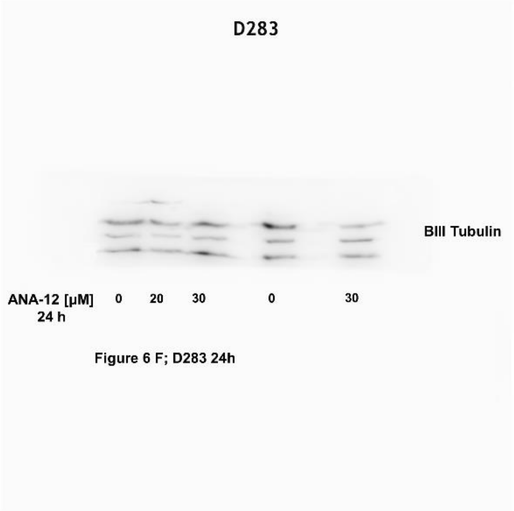

Figure 6 F; D283 24h

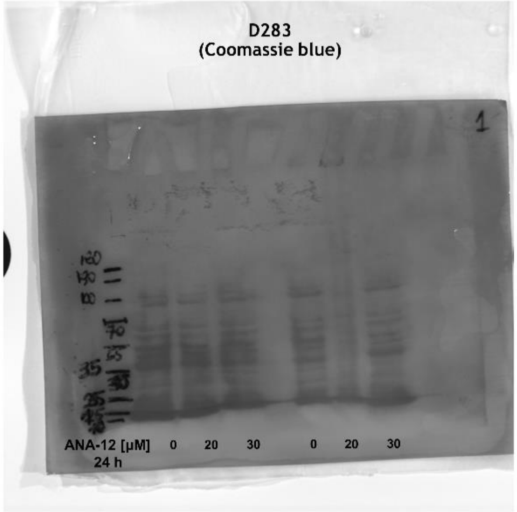

Supplement: Supplementary file 2 [file DataSheet_2.pdf]
